# Emphysematous cholecystitis in a young patient with no risk factors

**DOI:** 10.11604/pamj.2017.28.269.13923

**Published:** 2017-11-28

**Authors:** Dimitrios Manatakis, Grigorios Tsoukalos

**Affiliations:** 1 Department of Surgery, Athens Naval and Veterans Hospital, Athens, Greece; 2 Department of Interventional Radiology, Athens Naval and Veterans Hospital, Athens, Greece

**Keywords:** Gallbladder, acute Cholecystitis, emphysematous cholecystitis

## Image in medicine

A 43-year-old, otherwise healthy, male patient presented at the emergency department, with a 3-day history of right upper quadrant abdominal pain and fever of 39°C. Physical examination revealed positive Murphy’s sign and jaundice. Laboratory values revealed leukocytosis (18,000/ml), elevated CRP and liver function tests (direct bilirubin, ALP, γGT). Ultrasound scanning demonstrated distention of the gallbladder, with wall thickening, pericholecystic fluid and a gallstone impacted in cystic duct. The patient was empirically started on broad-spectrum antibiotics. An abdominal CT scan on the following day revealed gas in the gallbladder wall and lumen (A, B). A percutaneous cholecystostomy was placed (C) and his clinical condition improved dramatically over the following hours, with normalisation of inflammatory indices and liver chemistry. He is scheduled to undergo laparoscopic cholecystectomy in 8 weeks. Emphysematous cholecystitis usually begins as a typical case of acute cholecystitis, but ischemia and gangrene soon develop in the gallbladder wall, due to intramural translocation of gas-forming microorganisms (*Clostridium, E. coli, Klebsiella, Streptococci*). Predisposing factors include diabetes mellitus, immunosuppression, peripheral vascular disease, abdominal surgery and trauma. Initial assessment with ultrasound may show reverbation artifacts due to the presence of air. CT scans are considered more specific and sensitive, demonstrating a gaseous halo around the gallbladder as well as air within its lumen. Emphysematous cholecystitis is a surgical emergency. Early intervention with surgery or percutaneous cholecystostomy is the mainstay of therapy. Left untreated, it can progress to perforation, pericholecystic abscess or bile peritonitis. Mortality rate for uncomplicated emphysematous cholecystitis is 1.4%, whereas for complicated cases it can reach up to 25%.

**Figure1:**
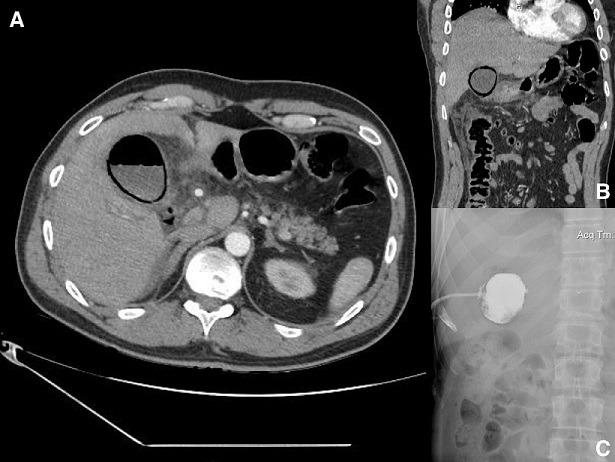
A) CT scan, axial, air within the gallbladder wall and lumen; B) CT scan, coronal, gaseous halo in the gallbladder wall; C) percutaneous cholecystostomy, complete obstruction of the cystic duct

